# Cancer cell resistance to anoikis: MUC1 glycosylation comes to play

**DOI:** 10.1038/cddis.2017.363

**Published:** 2017-07-27

**Authors:** Lu-Gang Yu

**Affiliations:** 1 Gastroenterology Unit, Department of Cellular and Molecular Physiology, Institute of Translational Medicine, University of Liverpool, Liverpool L69 3GE, UK

Anoikis is a special form of apoptotic process that occurs in cells in response to loss of adhesion to the extracellular matrix.^1^ It is a fundamental cellular process for maintaining tissue homoeostasis by removing displaced epithelial/endothelial cells and preventing them from seeding to inappropriate sites.

Anoikis activation begins from the cell surface through activation of the cell surface anoikis-initiating molecules such as integrins, cadherins and death receptors. Loss of cell adhesion to the extracellular matrix induces changes (e.g., conformation, dimer-/oligomer-ization or ligation with ligands) of the cell surface anoikis-initiating molecules that initiate a series of events leading to activation of either the caspase-8-mediated extrinsic apoptosis signalling or the mitochondrial-mediated intrinsic apoptosis signalling and consequent cell death.^2, 3^ For example, loss of engagement of cell surface integrin with the extracellular matrix induces integrin oligomer formation that leads to activation of the mitochondrial-mediated intrinsic apoptosis signalling. Loss of cell contact with the extracellular matrix induces ligation of the cell surface death receptors with their ligands in the surrounding, leading to activation of caspase-8-mediated extrinsic apoptosis signalling. Resistance to anoikis is a hallmark of oncogenic epithelial–mesenchymal transition and contributes prominently to tumorigenesis^4^ and, in particular, to metastasis by allowing survival of cancer cells that have invaded into the blood or lymphatic circulation and thus facilitating their metastatic spread to remote sites.

In 2014, we reported that expression of the transmembrane mucin protein MUC1 plays an important role in epithelial cancer cell resistance to anoikis.^5^ MUC1 is a very large (up to 500 kDa of molecular weight) and heavily glycosylated mucin protein that is ubiquitously expressed in all normal epithelial cells. MUC1 consists of a large and heavily glycosylated (up to 50% of the MUC1 molecular weight, mainly O-linked mucin type glycans) extracellular domain, a transmembrane region and a short cytoplasmic tail.^6^ In normal epithelium, MUC1 is expressed exclusively on the apical side of the epithelia and protrudes up to 10 times further above the cell surface than other typical cell surface molecules.^7^ In epithelial cancer, however, MUC1 expression is substantially increased (up to 10-fold), where it also loses its apical polarization and becomes expressed over the entire cell surface. Overexpression of MUC1 is closely associated with high metastatic potential and poor prognosis in cancer patients.^8^ Immunological targeting of cancer-associated MUC1 has been under intensive investigation as a strategy for cancer treatment.^9^ Both the MUC1 intracellular and extracellular domains were found in the earlier study to contribute to resistance of epithelial cancer cells to anoikis, but a pre-dominate influence was seen to come from the MUC1 extracellular domain.^5^

In a recent article published in *Cell Death Discovery*, shRNA suppression of the Core 1 Gal-transferase (C1GT, T-synthase) provides further insight into the action of MUC1 on epithelial cancer cell resistance to anoikis.^10^ C1GT is a key glycosyltransferase in the biosynthesis of O-linked mucin-type glycans.^11^ It is responsible for the formation of the Core 1 mucin-type carbohydrate structure (Gal*β*1,3GalNAc*α*-, Thomsen-Friedenreich, T or TF antigen),^12^ which acts as a base for further sugar chain elongation to form more complex (e.g., Core-2-associated) glycans.^13^ In this study, suppression of C1GT in three human colon cancer cell lines was shown to reduce the MUC1 *O*-glycosylation and substantially decrease the size of MUC1 extracellular domain (by >25%). This change significantly reduced the ability of the cancer cells to initiate anoikis in response to anoikis culture (suspension culture) of only the MUC1-positive cells but not the MUC1-negative cells. It showed that reduction of the MUC1 *O*-glycosylation exposed many cell surface anoikis-initiating molecules such as integrins, E-cadherin and Fas which otherwise were concealed by the large and heavily glycosylated MUC1 extracellular domain. Introduction of exogenous Fas-L showed to increase caspase-8 activity and anoikis in the MUC1-positive but not negative cells in comparison to their non-C1GT-suppressed control cells. These discoveries suggest an important role of MUC1 *O*-glycosylation in MUC1-mediated epithelial cancer cell resistance to anoikis.

The extracellular matrix contains huge amount of large and heavily glycosylated proteins (e.g., laminins, fibronectins).^14^ In normal epithelium, many of these large extracellular matrix glycoprotein interact with cell surface anoikis-initiating molecules (e.g., fibronectin interacts with integrins). Disengagement of these extracellular matrix glycoproteins with the anoikis-initiating molecules during cell detachment from the extracellular matrix likely represents a key sensor/trigger for conformation change and activation of the cell surface anoikis-initiating molecules in anoikis activation. MUC1 extracellular domain shares two characters with these extracellular matrix glycoproteins, it is large in size and is also heavily modified by complex carbohydrate structures. It is likely that the dense carbohydrate structures on MUC1 extracellular domain in epithelial cancer cells may provide a similar ‘home’ microenvironment to the cell surface anoikis-initiating molecules, as that provided by the extracellular matrix glycoprotein in normal epithelium, and thus prevent activation of these anoikis-initiating molecules during cell detachment from the extracellular matrix (Figure 1). This action of MUC1 also implies that other family members of the transmembrane mucin proteins (e.g., MUC4, MUC16 and so on), which are also huge in size and heavily glycosylated with dense O-linked glycans on their extracellular domains, may have similar influence on anoikis of epithelial cancer cells as MUC1. This study highlights the importance of cellular glycosylation in cell social behaviour and activity and provides a good example of the functional significance of cell surface glycosylation changes, commonly occurred in all cancer cells, in cancer development and progression.

## Figures and Tables

**Figure 1:**
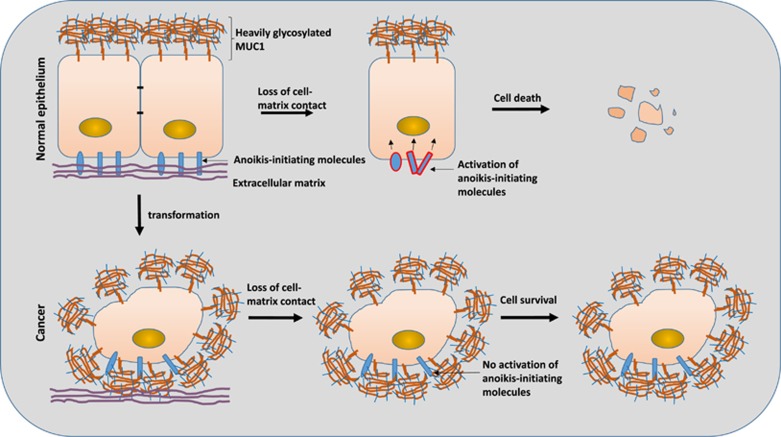
Model of the MUC1-mediated epithelial cancer cell resistance to anoikis. In normal epithelium, MUC1 is exclusively expressed on the apical side of the epithelia. Loss of cell contact with the extracellular matrix breaks interaction of the cell surface anoikis-initiating molecules with the heavily glycosylated extracellular matrix proteins. This results in conformation change and activation of these anoikis-initiating molecules, leading to apoptosis and cell death. In epithelial cancer cells, MUC1 is overexpressed over the entire cell surface and interacts with the cell surface anoikis-initiating molecules. The dense carbohydrate structures on MUC1 extracellular domain provides a ‘home’ microenvironment to the anoikis-initiating molecules, similar to that provided by the extracellular matrix glycoproteins in normal epithelium, and thus prevent their activation and cell death upon loss of cell–matrix contact
